# Custom-made hinged total knee arthroplasties in the context of extra-articular deformity: a case series

**DOI:** 10.1007/s00590-022-03299-8

**Published:** 2022-06-14

**Authors:** Timo K. Nuutinen, R. Madanat, K. W. Både, L. H. Ristolainen, H. Kauppinen, M. J. Manninen

**Affiliations:** 1 Orton Orthopaedic Hospital, Tenholantie 10, 00280 Helsinki, Finland; 2 Musculoskeletal and Plastic Surgery department, Helsinki University Hospital, Helsinki, Finland

**Keywords:** Custom-made, Knee surgery, Arthroplasty, Deformity

## Abstract

**Purpose:**

Treatment of secondary knee osteoarthritis with a significant extra-articular deformity can be challenging. In such cases, an osteotomy or a custom-made hinged knee arthroplasy (CMH) are treatment options. However, there are limited data on the outcomes of using CMHs. Thus, the aim of this retrospective study was to assess the clinical results and subjective outcomes of CMHs.

**Methods:**

We reviewed 9 CMHs (Endo-Model, LINK) in 7 patients with a minimum of 2-year follow-up. Upon the last follow-up, we evaluated MA, stability and range of movement (ROM). Oxford Knee Score (OKS) was used to evaluate patient-reported outcomes.

**Results:**

The average age upon surgery was 61 years (48–76 years), and the follow-up period was 66 months. There were no early complications. Two CMHs were revised, one due to aseptic loosening and one due to late-onset haematogenic infection. Pre-operatively, MA varied from 18° (average 11°) valgus-deformity to 30° (average 17°) varus-deformity. Post-operatively, 7/9 (78%) of patients achieved better MA. Upon follow-up, the average OKS was 41/48, and ROM was 113°.

**Conclusions:**

Patients treated with CMHs achieved good clinical and patient-reported outcomes. There were no early reoperations, and revision rate was relatively low. Overall, CMH could be considered for low-demand patients with increased operative risks.

## Introduction

Extra-articular deformity of the lower limb can be the result of a fracture malunion, a metabolic disorder, or a congenital abnormality. Extra-articular deformities may be located in the tibial or femoral diaphysis or metaphysis. Deformities can lead to abnormal mechanical alignment (MA) of the lower limb, which, in turn, may increase the risk of knee osteoarthritis (OA) [1]. When planning total knee arthroplasty (TKA), deformities should be taken into consideration to ensure better end results [2].

A deformity near the knee joint (e.g. metaphyseal deformity) has higher effect on the overall mechanical alignment compared to similar deformity further from the knee joint (e.g. diaphyseal deformity). The alignment can be corrected by performing TKA with maximal intra-articular resections or by performing simultaneous osteotomy [3]. Wang and Wang presented that maximal intra-articular resections produce a viable outcome if the proposed femoral resections are not more proximal than the collateral insertions, and the line from the centre of the talus and distal shaft of the tibia bypasses the tibial plateau [4]. New technologies, such as computer-assisted surgery and patient-specific instrumentation, might be helpful when performing maximal resections in contexts of extra-articular deformity [5–7].

In cases in which intra-articular resections are not sufficient, an osteotomy and TKA should be considered [3]. Several studies have reported good outcomes from simultaneous osteotomy and TKA, but also high risks of complications, such as perioperative fractures, non-unions and infections [8–13]. In some cases, in which constrained TKA is needed, deformities might inhibit standard stems from fitting in the deformed femur or tibia. In such cases, CMH might be used, but there is currently a lack of evidence supporting this technique [14, 15].

The aim of this retrospective multi-centre case series was to assess the mid-term clinical results and subjective outcomes of CMHs (Endo-Model, LINK, Hamburg, Germany) used in patients with secondary osteoarthritis and associated deformity of the lower limb. CMH is technically easy to perform; it also allows immediate full weight bearing and features no risk of non-unions. However, there have been problems with hinge mechanism over time such as high rates of aseptic loosening. Thus, we hypothesized that, in high-risk patients or in cases in which standard stems do not fit in the femur or tibia, CMH could produce decent outcomes with a reduced risk of complications.

## Materials and methods

### Patients and outcomes

Since 2003, fourteen CMHs (Endo-Model, Link, Hamburg) have been implanted in Finland for secondary knee OA in difficultly deformed bony anatomy. We excluded five CMHs from our study, as they were used in revision surgery. Thus, nine CMHs used in seven patients, with a minimum of 2-year follow-up, were included. Two out of seven patients had undergone bilateral CMH operations. The operations were performed in four hospitals. Prophylactic antibiotic (kefuroxime or clindamycin) was used just before the operation. Post-operatively full weight bearing and free mobilization were allowed.

We recorded patients’ previous medical history for demographic data and assessed their radiographs. We contacted patients via telephone, and they were invited for a follow-up visit. At this follow-up visit, we examined the stability and range of movement (ROM) of the knee, and patients were asked to complete the Oxford Knee Score (OKS, maximum is 48 points) questionnaire for the affected knee. Two authors (T.N. and M.M) evaluated and measured their pre- and post-operative MA from anteroposterior weight-bearing radiographs, and radiolucent lines from native knee radiographs. Any complication or revision was noted.

### Custom-made hinged total knee arthroplasties

**Fig. 1:**
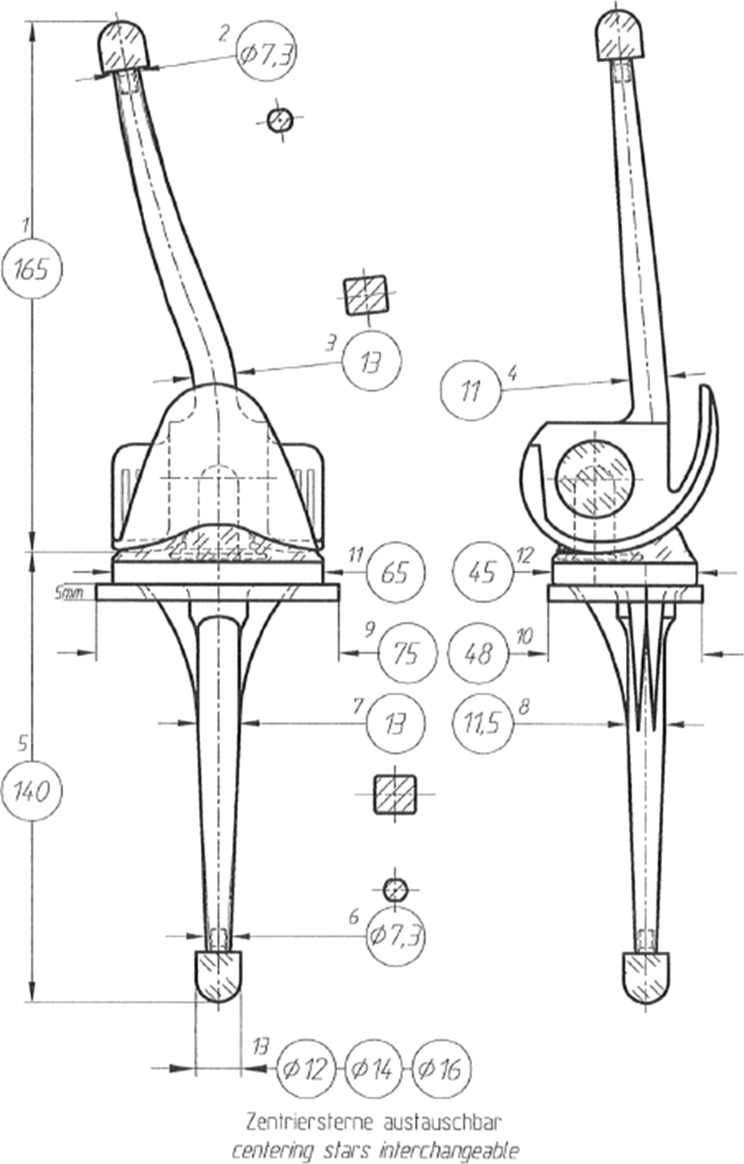
Figure of the template of the implants

All CMHs were customized Endo-Model (LINK, Hamburg), which is a third-generation hinged TKA [16]. In the normal Endo-Model, the femoral component has 6° of valgus. Implants were cobalt–chromium–molybdenum alloy, and the tibial plateaus were UHMW polyethylene. The CMH implants were customized based on pre-operative native X-rays and computed tomography. The stem offset, angles and curves were customized so that the stems would fit well in the deformed femur and tibia. The other aim was to correct the MA better than it was in the anatomical extremity. The customization of each case is shown in Table 1, and an example of the prosthesis template is shown in Fig. 1.

**Table 1:** Patients’ demographic data

Patient	Age (at surgery)	Gender	Side	Diagnosis (of deformity)	Side of the deformity	Previous surgeries	Prothesis
1	50	F	R	Osteogenesis imperfecta, oligoarthritis	Tibial and femoral diaphyseal	ACL-reconstruction left	Femur and tibia anatomically curved
1	50	F	L	Osteogenesis imperfecta, oligoarthritis	Tibial and femoral diaphyseal	ACL-reconstruction left	Femur and tibia anatomically curved
2	70	F	L	Bone tuberculosis and hip dysplasia	Femoral metaphyseal	Subtalar arthrodesis, ACL-reconstruction, THA left	Femur and tibia anatomically curved
3	48	F	L	Juvenile rheumatoid arthritis	Tibial diaphyseal	Epiphysiodesis both, Osteotomy and TKA right, THA both	Femur stem anatomically curved, tibia stem medialized
4	61	F	R	Hypophosphatemia	Femoral diaphyseal	–	Femur stem anatomically curved and shortened, tibia stem shortened and proximal tibia spacer
4	60	F	L	Hypophosphatemia	Femoral diaphyseal	–	Femur stem anatomically curved and shortened, tibia stem shortened
5	71	M	R	Patella and growth plate fracture at age 11 and tibia fracture at age 28	Tibial diaphyseal	Double osteotomies, ORIF of fibula, and several debridements of the right knee	Tibia stem anatomically curved and proximal tibia spacer
6	66	F	L	Unknown skeletal disorder that has lead curved tibia	Tibial diaphyseal	Medial meniscus resection left	Tibia stem anatomically curved and lateralized
7	76	M	R	Femur fracture at age 22	Femoral diaphyseal	–	Femur stem anatomically curved

*THA* total hip arthroplasty, *ORIF* open reduction and internal fixation, *ACL* anterior cruciate ligament, *TKA* total knee arthroplasty

### Data analysis

IBM SPSS Statistics (version 28.0.1) was used to carry out descriptive analyses. Pair sample *t* test was applied to calculate statistical differences between pre-operative and post-operative variables. The statistically significant threshold was accepted at *p* ≤ 0.05 (two-tailed).

### Ethical approval

The study was approved by the ethical committee of Helsinki University Hospital. Informed consent was obtained for each patient in the study.

## Results

### Demographics

The average age of the patients undergoing the index operation was 61 years (48–76 years), and 5/7 (71%) were women. The causes of lower-limb deformity were previous trauma (*n* = 2), osteogenesis imperfecta (*n* = 2), hypophosphatemia (*n* = 2), juvenile rheumatoid arthritis (*n* = 1), previous bone tuberculosis (*n* = 1) and an unknown skeletal disorder (*n* = 1). Two patients had undergone treatment with bilateral CMHs. The average follow-up time was 66 months (24–97 months). The average pre-operative Kellgren–Lawrence osteoarthritis classification was 3.4 (3–4).

The average pre-operative flexion was 103° (80°–135°). An extension deficit was present in three knees, and three knees went into hyperextension. Post-operatively, ROM varied from 70° to 135°, and the average ROM was 113°. Six out of nine (57%) of the knees achieved the same or better ROM post-operatively. One patient had 10° lack of extension.

At the last follow-up visit, the average OKS was 41. Moreover, two patients limped at this time: one clearly and one mildly. In one knee, there was slight movement in the coronal plane upon clinical assessment and radiolucent lines (maximum of 3.7 mm) around the femoral component. All other knees were stable in both the anteroposterior and coronal planes, and no signs of aseptic loosening were observed.

### Mechanical alignment

Pre-operatively, MA varied from 18 (an average of 11) degrees of valgus-deformity to 30 (an average of 17) degrees varus-deformity. Post-operatively, mechanical alignment varied from 16 (an average of 7) degrees of valgus to 9 (an average of 4) degrees of varus. In seven (77%) cases, MA was closer to neutral post-operatively than pre-operatively. In two cases, valgus-deformity was higher post-operatively (from 12 to 15 and 8 to 9 degree of valgus).

### Complications

After operation, no immediate complications occurred. Revision surgery was performed for 2/9 (22%) of the CMHs during the follow-up period. One patient had pain in the right knee and signs of aseptic loosening around the femur component. The femur component was exchanged 3 years after the primary operation. In the last follow-up, after 8 years from primary operation, there were signs of aseptic loosening around the same femur component, as mentioned above, but at this time did not require revision surgery. Another patient with previous bone tuberculosis had a haematogenic infection of the implant 4 years after the CMH operation. This infection was treated with antibiotics and debridement of the knee, and the liner was exchanged (DAIR). Meanwhile, neither tibial nor femoral bone components were exchanged.

## Discussion

The overall mid-term subjective outcomes of CMH operations were encouraging. Patients exhibited relatively good ROM and stability with high patient-reported outcomes. CMH seems to be safe option for low-demand and high-risk patients, as there were no early complications. Otherwise, post-operative alignments were not as good as intended.

Several studies have evaluated the outcomes and survivorship of the Endo-Model hinged prosthesis [17–22]. Lombardi et al. demonstrated a survival of 85% in a series of 109 patients (113 TKAs), with a mean 25-month follow-up period [20]. Sanguineti et al. evaluated 20 knees for a mean duration of 42 months and reported a survival of 95% [22]. As with other hinged implants, the most commonly reported complications have been aseptic loosening and deep infection—as like in our series [19, 22]. There are only a few published studies related to the use of a CMH in patients with lower-limb deformity or neuromuscular dysfunction [14, 23]. In these studies, the prothesis used was the Stanmore Modular Individualized Lower Extremity System prosthesis. Sewell et al. reported good relief of pain and functional outcomes in 11 patients with skeletal dysplasia treated with CMH [14]. There was one (9%) aseptic loosening of the femur component and three other (one patella fracture, one tibia fracture, and one patella impingement) complications during an average seven-year follow-up time. Compared to Sewell’s study, in our series, the complication rate was somewhat lower (Table 2).

**Table 2:** Patients’ outcomes

Patient	Post-operative OKS	Pre-operative ROM	Post-operative ROM	Pre-operative MA^a^	Post-operative MA^a^	Change of the MA^b^	Revisions	Radio lucent lines
1	39	Hyper extension—120	0–110	− 12	− 16	− 2	Aseptic loosening → Femur component	Yes
1	42	Hyper extension—120	0–120	8	− 6	14	No	No
2	38	0–80	10–80	− 8	− 9	− 1	Haematogenic infection → DAIR	No
3	43	0–90	0–110	− 3	− 1	2	No	No
4	46	20–90	0–135	30	9	21	No	No
4	46	30–80	0–135	16	2	14	No	No
5	46	Hyper extension—90	0–110	− 18	− 9	9	No	No
6	45	0–120	0–123	13	0	13	No	No
7	21	5–135	0–110	− 15	− 2	13	No	No

*OKS* Oxford knee score, *ROM* range of movement, *MA* mechanical alignment, *NA* not available

^a^Positive number presents varus-deformity and negative number valgus-deformity

^b^Positive number presents correction towards neutral and negative towards

Previous studies have also reported good outcomes when correcting for extra-articular deformity with intra-articular resections or simultaneous osteotomy. However, intra-articular resections are not always sufficient and radical resections may lead to difficulties in balancing without constrained implant, and simultaneous osteotomy should be considered when performing TKA [3, 4, 8–11, 13]. Since simultaneous osteotomy is jeopardized by high rates of complications and the fact that standard stems may not always fit in a deformed tibia or femur, our aim was to identify a solution for this rare patient group using CMHs, even though we were aware of the complications of using fully-constrained implant as mentioned above.

**Fig. 2:**
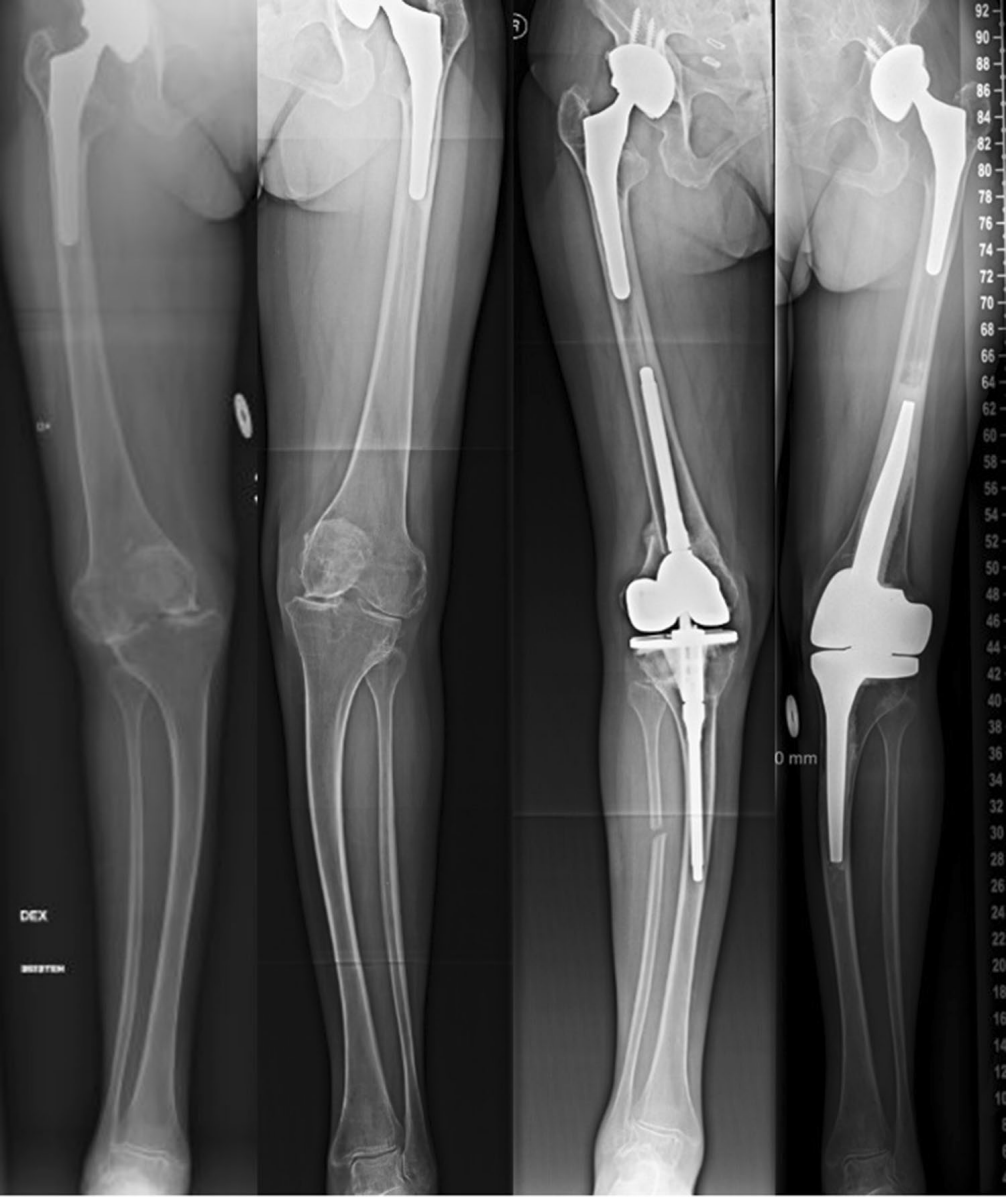
Patient with juvenile rheumatoid arthritis. She had osteotomy and conventional hinged TKA on right side and later CMH on left side. Recovery was easier and sick leave shorter after the CMH operation

In our study, no immediate complications occurred with the CMH. In previous studies (Table 3), which have evaluated patients with simultaneous osteotomy and TKA, the incidence of delayed union or non-union has been up to 15% [11–13, 24]. Moreover, rates of infection, periprosthetic fractures, thromboembolic complications, and stiffness have been even higher [8–11, 13]. The CMH operation is relatively easy to perform, and patients can achieve full weight bearing immediately, which may decrease thromboembolic complications [25]. In this study, we did not have a control group, but interestingly, one patient with juvenile rheumatoid arthritis had had osteotomies of both the femur and tibia, a simultaneous conventional hinged TKA in the right knee. Pre- and post-operative radiographs are shown in Fig. 2. A few years later, a CMH was implanted in her left knee. This patient reported that recovery was much easier on the left side, and her sick leave duration was two-third shorter after the CMH operation when compared to those related to her earlier osteotomies and TKA procedure on the right leg. Furthermore, customization enables using a hinged prothesis despite the presence of a deformity that prevents standard stems fitting femur or tibia.

**Table 3:** Literature review of simultaneous osteotomy and TKA

Study (year)	*N*	Follow-up (months)	Deformity	Technique	Results	Complications
Lonner et al. [11]	11	46	Varus ≥ 14°or ≥ 25° antecurvatum	FO + TKA	KSS 10 → 87	18%
						1 pulmonary embolism
						1 non-union
Radke et al. [24]	10	30	Varus or valgus > 15°	TO + TKA	KSS 28 → 81	20%
						1 DVT
						1 delayed union
Madelaine et al. [12]	15	78	Varus or valgus > 10°	TO + TKA	KSS 47 → 61	53%
						4 perioperative fracture
						2 non-union
						1 deep infection
						1 stiffness
Veltman et al. [13]	21	64	Average 14° varusor 12° valgus	TO/FO + TKA	OKS 39	18%
						2 deep infection
						2 non-union
						1 fracture
						1 stiffness
Demir et al. [10]	12	44	Varus or valgus ≥ 30°or severe rotation	TKA followed by osteotomy	OKS 9 → 42	42%
						1 malposition
						1 perioperative fracture followed by deep infection
						3 “butterfly component”
Catonne et al. [8]	6	120	Varus ≥ 18°or severe rotation	FO + TKA	IKS 46 → 161	33%
						1 DVT
						1 stiffness
Catonne et al. [9]	26	108	Varus or valgus ≥ 11°, severe translation, recurvatum, or flexion	TO + TKA	IKS 70 → 170	23%
						4 fracture
						1 DVT
						1 infection and necrosis

*TO* tibial osteotomy, *FO* femoral osteotomy, *DVT* deep venous thrombosis, *KSS* Knee Society Score, *IKS* International Knee Society knee and function score, *OKS* Oxford Knee Score

**Fig. 3:**
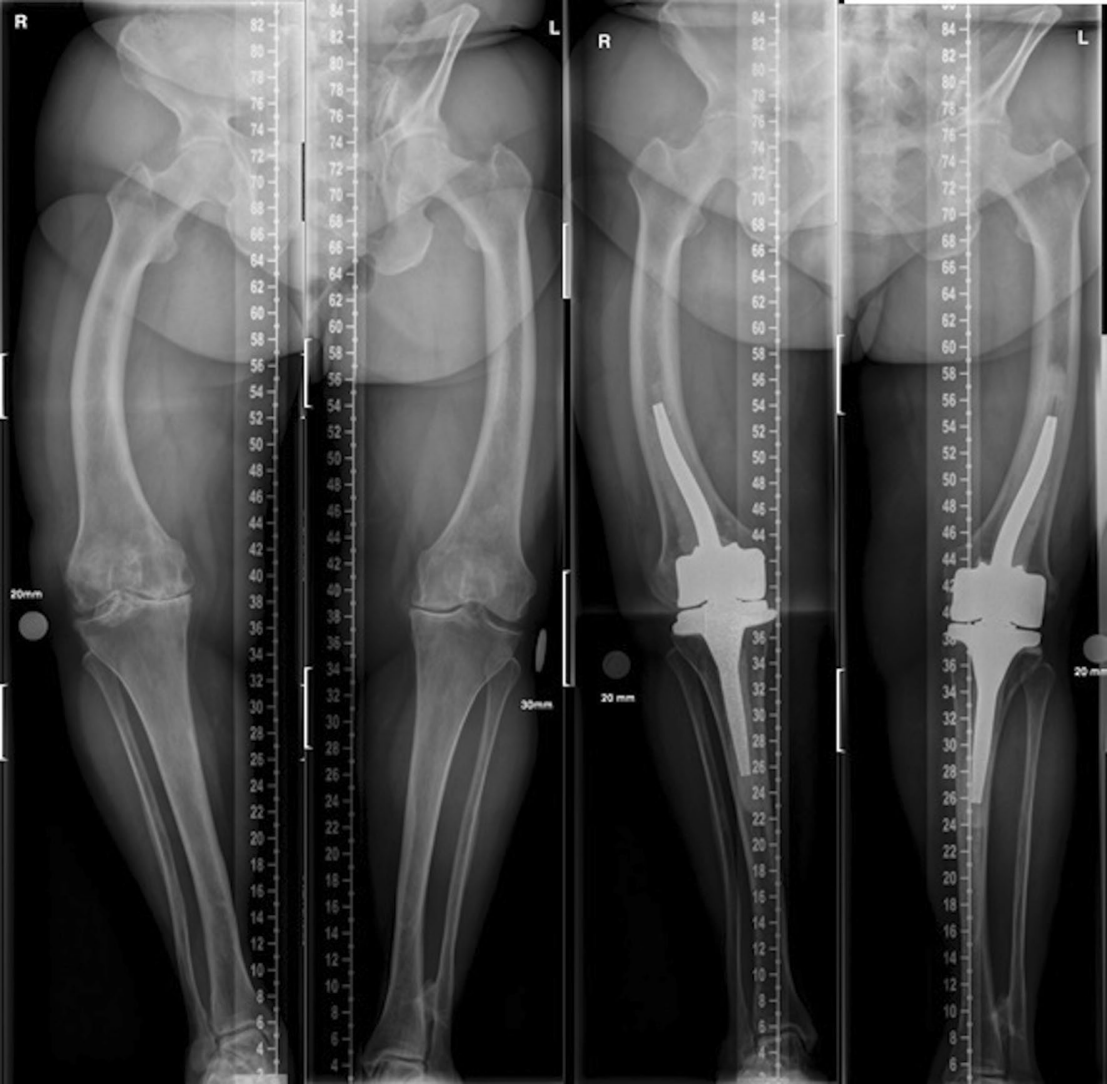
Familial hypophosphatemia patient. Pre- and post-operative long leg weight-bearing radiographs of a patient with familial hypophosphatemia. Pre-operatively, osteotomy and TKA were considered, but, due to an increased risk of complications (diabetes, smoker), the surgeon decided to first perform CMH on the right knee. The patient was pleased with the outcome, and the left knee was also operated upon five months later using the same technique. The post-operative MA was 9 (right) and 2 (left) degrees of varus

Compared to osteotomy and TKA, the CMH seemed to exhibit poorer MA post-operatively [8, 9, 11, 12, 24]. In the present study neutral (0 ± 5 degrees), MA was achieved in 4/9 cases. Two authors (T.N. and M.M.) evaluated the radiographs, and in the cases where neutral MA was not achieved, technical prothesis implantation error was not found. It was thought that easily when planning the CMH the attention has probably been on fitting of the stems in curved bones. The pre-operative planning by surgeons and manufacturer had not taken enough into account the deformities and pre-operative MA—tibial and/or femoral valgus-angles of the stems were not sufficient to fully correct the deformity of the whole leg as shown in Fig. 3. For example, one patient (case #1) with post-operative 16° of valgus had aseptic loosening, and in this case, femoral and tibial stems were bent to fit the bones, but the angle of the stems was not sufficient to fully correct the MA. Therefore, more attention should be paid to the pre-operative planning process.

**Fig. 4:**
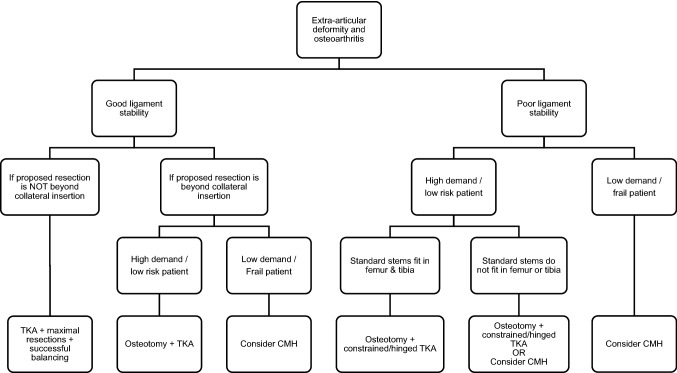
The treatment algorithm. The proposed treatment algorithm for patients with a severe extra-articular deformity and secondary osteoarthritis

Even though there were problems with the post-operative MA and relatively high rate of aseptic loosening, we think that CMHs could be considered as an option for high-risk and low-demand patients with a severe extra-articular deformity and a secondary osteoarthritis. But due to relatively high risk of aseptic loosening, it should not be the first-line option for young and active patients. Our proposed treatment algorithm is in Fig. 4.

**Fig. 5:**
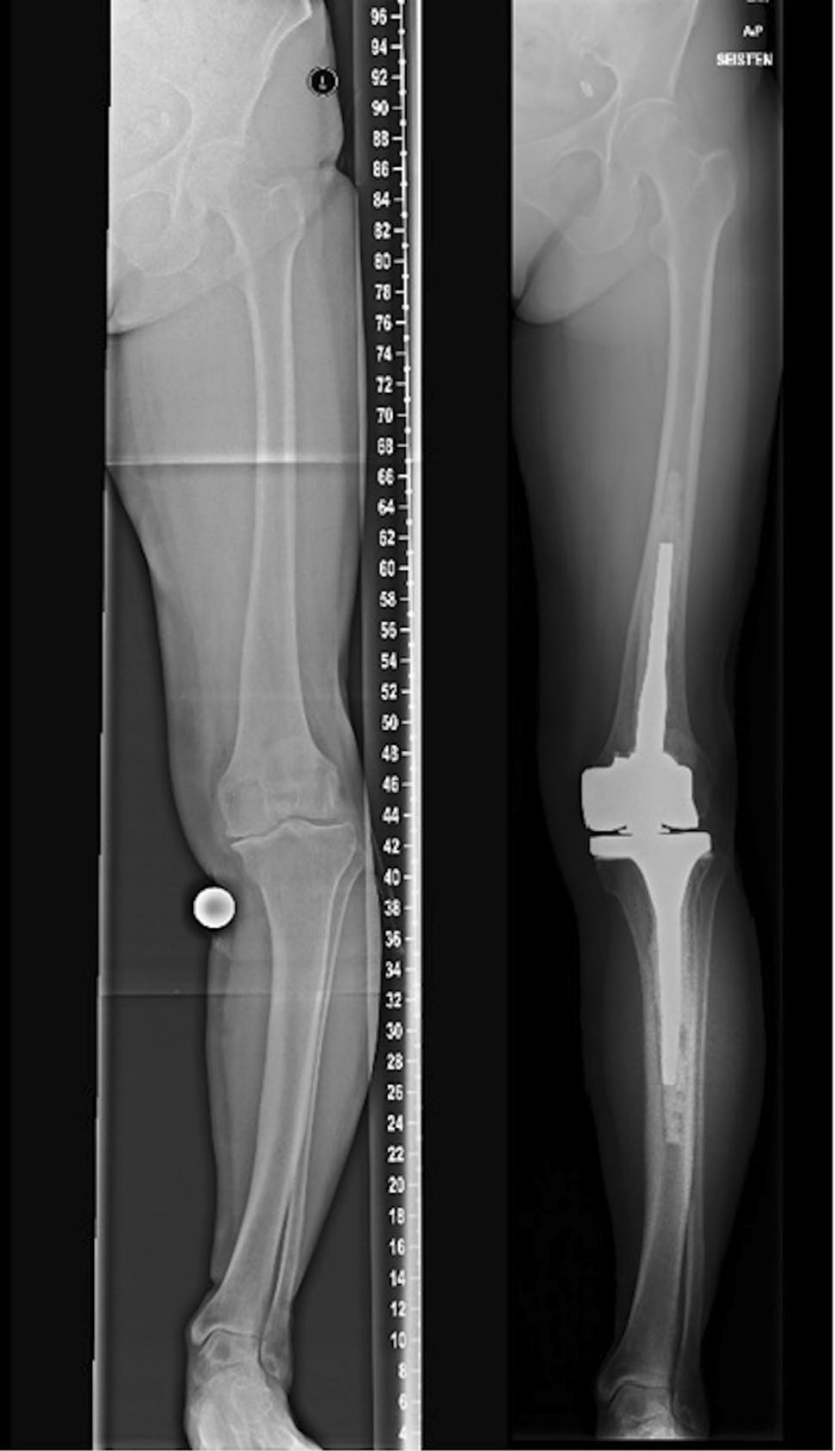
Patient with unknown skeletal disorder. Pre- and post-operative long leg weight-bearing radiographs of a patient with an unknown skeletal disorder that led to a curved tibia. The surgeon considered other options, such as patient-specific instrumentations, but ended up deciding on a CMH. MA was corrected to normal, and OKS was 45

We are aware that our study has several limitations. Specifically, the retrospective study design has inherent limitations, and, due to the nature of the study, there was no control group. Patients were also a heterogenic group in terms of previous surgical procedures, deformity, and diseases. And since the rarity of these extreme deformities, the sample size is limited. We did not register use of tourniquet, blood loss or duration. Results are also valid only for this implant. Nevertheless, our study provides valuable novel information on the planning and use of the CMH in the treatment of these very rare and complex clinical scenarios, for which a prospective study is not feasible (Fig. 5).

## Conclusions

In our study, patients with extremely deformed lower limb achieved good clinical and patient-reported outcomes. There were no early reoperations. Overall, CMH could be considered especially for low-demand patients with an increased operative risk.
